# The application of an allogeneic bone screw for osteosynthesis in hand and foot surgery: a case series

**DOI:** 10.1007/s00402-021-03880-6

**Published:** 2021-04-08

**Authors:** Klaus Pastl, Wolfgang Schimetta

**Affiliations:** 1 Orthopädie Klinik Diakonissen Linz, Weißenwolfstraße 15, 4020 Linz, Austria; 2 Department of Applied Systems Research and Statistics, Johannes Kepler University, 4040 Linz, Austria

**Keywords:** Allogeneic bone screw, Shark Screw® transplant, Osteosynthesis, Osteotomy and arthrodesis, Hand and foot surgery, Allograft

## Abstract

**Introduction:**

The allogeneic bone screw transplant is a new osteosynthesis device making the use of foreign fixation material obsolete for various kinds of indications. Moreover, it is integrated into the recipient bone by natural bone remodeling without harming the surrounding tissue. The aim of this study was to determine the efficacy and safety of the transplant for osteotomy and arthrodesis in hand and foot surgery and to evaluate the clinical importance of the device.

**Materials and methods:**

A single-surgeon case series of 32 patients who had undergone hand or foot surgery with the application of an allogeneic bone screw with an average follow-up time of 1 year is reported. Clinical data were reviewed to evaluate the pain levels and satisfaction of the patients and the frequency and type of complications occurring during the healing process. Routine radiography and computed tomography were reviewed to determine the fusion rate, the ingrowth behavior of the transplant and the possible occurrence of transplant failure.

**Results:**

High patient satisfaction was paired with low postoperative pain levels and a low complication rate. 97% of the patients were free of pain at the timepoint of the second follow-up examination, the mean time of recovery of full mobility was 50.1 ± 26.1 days after surgery. Wound healing disturbance occurred only in two cases. Bony consolidation of the osteotomy or arthrodesis gap as well as osseointegration of the transplant was seen in all cases. No transplant failure or transplant loosening occurred.

**Conclusions:**

The application of the allogeneic bone screw resulted in a 100% fusion rate while the patient burden was low. The transplant is safe and suited for various kinds of osteosynthesis in hand and foot surgery.

## Introduction

The Shark Screw® transplant (surgebright GmbH, Austria) is a human cortical bone allograft for osteosynthesis and an alternative to metal or bioabsorbable devices in orthopedics and trauma surgery. The use of this allogeneic screw allows fracture, osteotomy and arthrodesis fixation with a human bone transplant. The additional application of non-human material is fully dispensable. Besides the fixation function due to its design as a setscrew, the allogeneic screw exhibits osteoconductive properties promoting the ingrowth of blood vessels and bone cells [1]. It is integrated into the recipient bone by the continuous bone remodeling process, thereby leading to full conversion into autologous bone. This bone remodeling process does continuously occur within the bone and is not specifically triggered by the allograft.

In general, the biology of bone grafting is well studied. Allogeneic bone grafts are incorporated by creeping substitution, with an initial osteoclastic activity followed by vascularization of the graft tissue and subsequent bone remodeling [1–3]. The safety of allogeneic, sterilized bone transplants regarding disease transmission, the biological tolerance, potential graft rejection and allosensitization is well known since allogeneic bone transplants (e.g. bone chips, bone blocks) are widely used in regenerative, maxillofacial and orthopedic medicine [4–7].

The use of screws made of human bone has been described earlier by various authors. In 1957, Schwier successfully fixed tibia fractures with allogeneic bone screws [8]. Zaborszky and Hommel used allogeneic bone for the stabilisation of pseudarthrosis in 1967 [9, 10] and Grasser applied allogeneic cortical screws in oral and maxillofacial surgery in 1968 [11]. Between 1994 and 1998, reports on the clinical application of screw transplants made of allogeneic bone in oral and maxillofacial surgery, hand and foot surgery and the treatment of Osteochondritis dissecans have been published [12–15].

Currently used metal implants remain within the body as a foreign matter. They may provoke pain sensation, soft tissue irritations, allergic reactions or functional inhibition and hardware removal may become necessary [16–18]. Bioabsorbable osteosynthesis devices (e.g. biodegradable polymers or magnesium-based materials) may be used alternatively to metal devices. They do not have to be removed, yet these materials and their degradation products may interfere with bone healing and an inflammatory response may be triggered during degradation [19, 20]. Additionally, bioabsorbable materials must show a balanced degradation rate to provide stabilization throughout the bone healing process.

The Shark Screw® transplant is the first commercially available osteosynthesis transplant in Austria.

The aim of this study was to evaluate the efficacy and safety of the allogeneic screw in hand and foot surgery by analyzing the recovery and healing process. The advantages and drawbacks of the human bone transplant in hand and foot surgery should be assessed.

## Patients/methods

In this retrospective case series, 32 patients (27 female, 5 male) with an average age of 66.9 ± 10.9 years were included. All patients had undergone hand or foot surgery with the use of allogeneic screws for joint arthrodesis or osteotomies between October 2016 and January 2018. Ethical approval has been received from the ethics committee of Upper Austria (Vote-No: 1099/2018).

The mean follow-up time was 368 ± 57 days. This period of time is sufficient to assess the wound healing and recovery process, fusion rate and the bony consolidation of the transplant.

14 patients had undergone hand surgery and 18 patients were in the foot surgery group. All surgeries were done by the same surgeon. A detailed overview of all surgical interventions performed is shown in Table 1.

**Table 1:** Overview of surgical interventions included in this study

Hand surgeries	Number of surgical procedures performed
Distal interphalangeal joint arthrodesis	7
Four-corner fusion	3
Thumb interphalangeal joint arthrodesis	1
Thumb carpometacarpal joint arthrodesis	1
Combined proximal interphalangeal joint and distal interphalangeal joint arthrodesis	1
Combined thumb interphalangeal joint and distal interphalangeal joint arthrodesis	1
Total (Hand)	14

Foot surgeries	Number of surgical procedures performed
Metatarsophalangeal joint I arthrodesis	4
Austin osteotomy	4
Scarf osteotomy	2
Combined tarsometatarsal joint II and III arthrodesis	3
Lapidus arthrodesis	3
Austin V osteotomy	1
Interphalangeal joint arthrodesis of the big toe	1
Total (Foot)	18

50 allogeneic screws with different outer diameter and a constant length of 35 mm have been used. An overview of the size of transplants used is given in Table 2.

**Table 2:** Overview of allogeneic screws used in this study

Type of surgery	Allogeneic screw diameter (mm)				Total number of allogeneic screws
	3.5	4.0	4.5	5.0	
Hand surgical procedures	19	2	1	–	22
Foot surgical procedures	–	9	6	13	28

The insertion of the allogeneic screw was done following a standardized procedure. After cartilage removal, the articular or osteotomy surface was put under compression and temporarily fixed with a Kirschner wire, defining the final position of the allogeneic screw. Subsequently, core drilling and thread cutting was performed. Depending on the size of the recipient bone, the allogeneic screw with the largest diameter possible was chosen. After screwing in the transplant, the protruding part of the screw was removed planar to the level of the recipient bone. Postoperatively, the hand or foot was immobilized according to the surgeons discretion with initial non-weight bearing followed by a period of partial weight-bearing in case of foot surgery.

All clinical records, surgical reports, radiology, and computed tomography images of each patient served as data sources. The first, second, and third routine follow-up examinations took place 6 weeks (− 2/ + 3 weeks), 15 weeks (− 6/ + 15 weeks) and 1 year (− 3/ + 6 months) after surgery.

Data on the bone quality of the recipient bone, the primary stability of the osteosynthesis and the use of additional fixation material were collected from the surgery report. Bone quality was classified as “very good” if the bone substance did not show any osteoporotic changes or cysts. If there were partially osteoporotic changes in the bone substance, the quality was classified as “good”. The bone quality was rated as “moderate” if the bone was osteoporotic with ubiquitously declined bone substance. The stability of the osteosynthesis was classified as “very good” if no micromovements were detectable in the fracture gap. If micromovements were visible during surgery or the fracture gap surfaces were not closely attached to each other, the stability was classified as “good”. A higher degree of movement was classified as “moderate stability”.

Data on pre- and postoperative pain sensation were collected (visual analogue scale VAS [21]; 0 = no pain, 10 = worst imaginable pain). In addition, the duration of postoperative analgesic medication was assessed. Records were also reviewed regarding the time to recovery of full mobility and the incidences of revision surgery. Patient satisfaction was analyzed at the timepoint of the last follow-up examination and was classified into “very satisfied”, “satisfied”, and “not satisfied”. Moreover, the type and frequency of postoperative complications and soft tissue irritation was assessed.

The radiographs taken at each timepoint of examination were analyzed. Consolidation of the transplant was determined by the absence of radiolucent lines. A radiolucent line more than 2 mm wide, spanning more than 50% of the transplant circumference including the transplant tip and showing temporal dynamics points towards impaired ingrowth or missing bony consolidation. Small radiolucent areas less than 2 mm wide have been classified as unspecific. Sclerosis around the transplant indicates a lack of cellular contact to the recipient bone, whereas cystic lucency indicates resorptive processes in the recipient bone. Rupture or dislocation of the transplant with visible fissures indicates transplant overload.

Routinely, osseous fusion was evaluated by radiology if two orthogonal images were available. Osseous fusion was determined by analyzing the radiolucency in the arthrodesis or osteotomy gap. If not assessable on X-ray images, additional CT examination had been performed.

Statistical analysis was performed using the open-source R statistical software package, version 3.4.2 (The R Foundation for Statistical Computing, Vienna, Austria).

Nominally scaled data were calculated with absolute and relative frequencies and are presented as such. For quantitatively measured data minimum, median, quartiles, maximum, arithmetic mean and standard deviation were calculated. Data are presented as means plus minus standard deviations.

Missing values were not replaced with one exception. In case a patient took analgesic medication for more than 1 day within the last 7 days, the VAS value was set as implausible and the missing value was replaced according to the worst-case principle.

## Results

The primary stability of the osteosynthesis has been rated as “very good” in 24 cases (75.0%) and “good” in 8 cases (25.0%). In none of the cases the osteosynthesis has been rated as “moderate”. Additional fixation material was not used. The quality of the recipient bone was rated 14 times (43.8%) as “very good”, 6 times (18.8%) as “good” and 12 times (37.5%) as “moderate”.

The mean duration of analgesic treatment after surgery was 2.3 ± 5.0 days (*n * = 32). 14 patients (43.8%) did not need pain relief at all. The mean time to recovery of full mobility was 50.1 ± 26.1 days (*n * = 32).

The preoperative pain value according to VAS was 6.4 ± 1.1 (*n * = 32). At the timepoint of the first follow-up examination (43.1 ± 9.2 days after surgery), the mean pain value was 0.6 ± 1.3 (*n * =  30). In detail, 24 patients (80.0%) were free of pain (VAS = 0), 6 patients (20.0%) reported pain levels between 2 and 5. At the second postoperative examination (141.4 ± 46.5 days after surgery), 30 patients (96.8%) were free of pain (VAS = 0), only one patient indicated a VAS pain value of 2 (*n * = 31). At the last follow-up examination (368.4 ± 56.7 days after surgery), all patients (*n * = 28) were free of pain (VAS = 0). Table 3 shows the VAS pain values at the different timepoints of examination.

**Table 3:** VAS pain values at the various timepoints of examination

	VAS pain level ± standard deviation	Minimum value	Maximum value
Before surgery (*n* = 32)	6.4 ± 1.1	5	8
1st follow-up examination (*n* = 30)	0.6 ± 1.3	0	5
2nd follow-up examination (*n* = 31)	0.1 ± 0.4	0	2
3rd follow-up examination (*n* = 28)	0.0 ± 0.0	0	0

In total, 28 patients indicated to be “very satisfied” with the result at the last follow-up examination. For four patients records on satisfaction were not available.

**Fig. 1:**
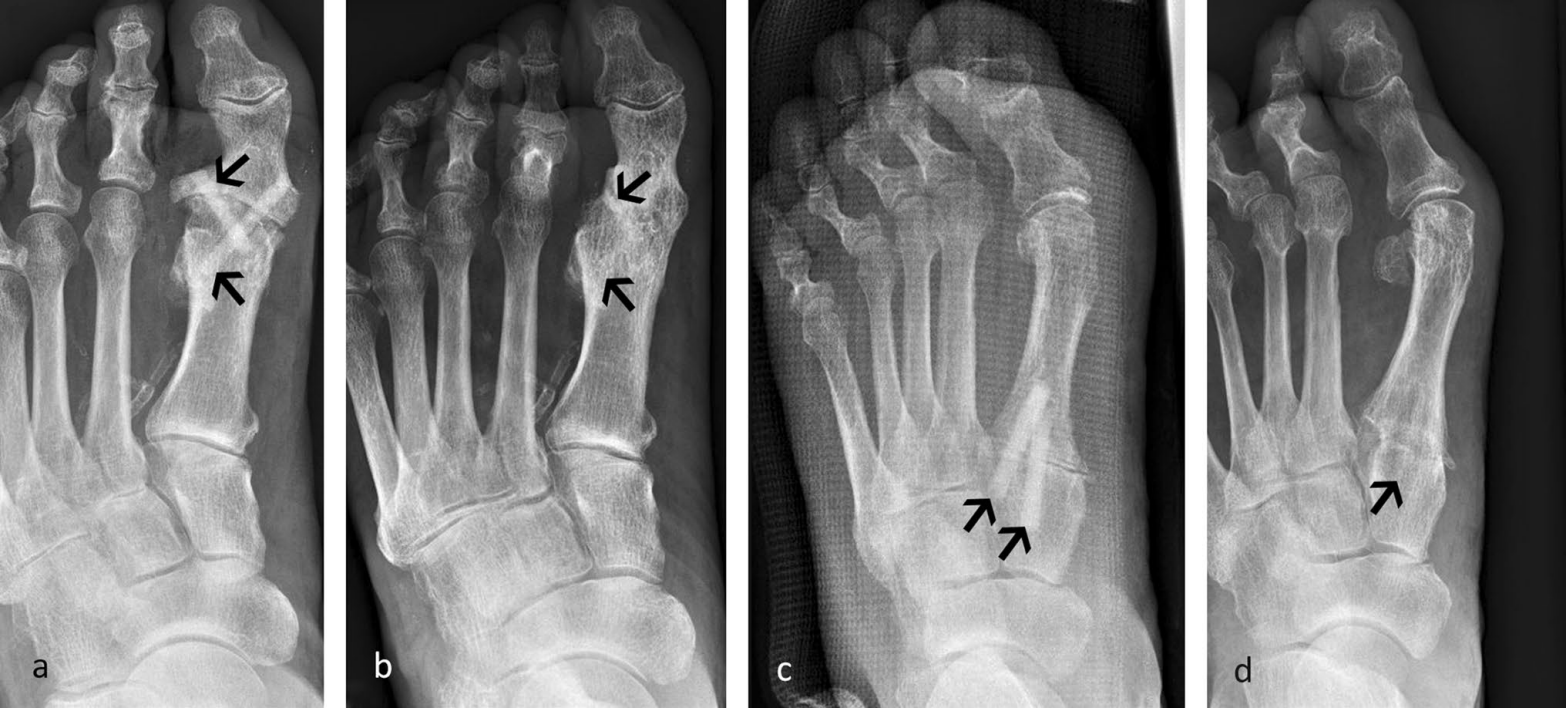
Examples of the application of the allogeneic screw in foot surgery. Radiographs showing the incorporation of the transplant used for an MTP I arthrodesis of a patient with type 2 diabetes and peripheral arterial occlusive disease **a** on the day of surgery, **b** 20 months after surgery and radiology showing the incorporation of the allogeneic screw used for a Lapidus arthrodesis **c** 6 weeks and **d** 12 months after surgery. Transplants are marked by arrows

Figure 1 shows a metatarsophalangeal (MTP) I joint arthrodesis (Fig. 1a, b) and a Lapidus arthrodesis (Fig. 1c, d) as examples of foot surgical procedures. In both cases, joint fusion and remodeling of the transplants is visible. The transplants are hardly visible on the radiographs 20 months after surgery (Fig. 1b) and 12 months after surgery (Fig. 1d).

**Fig. 2:**
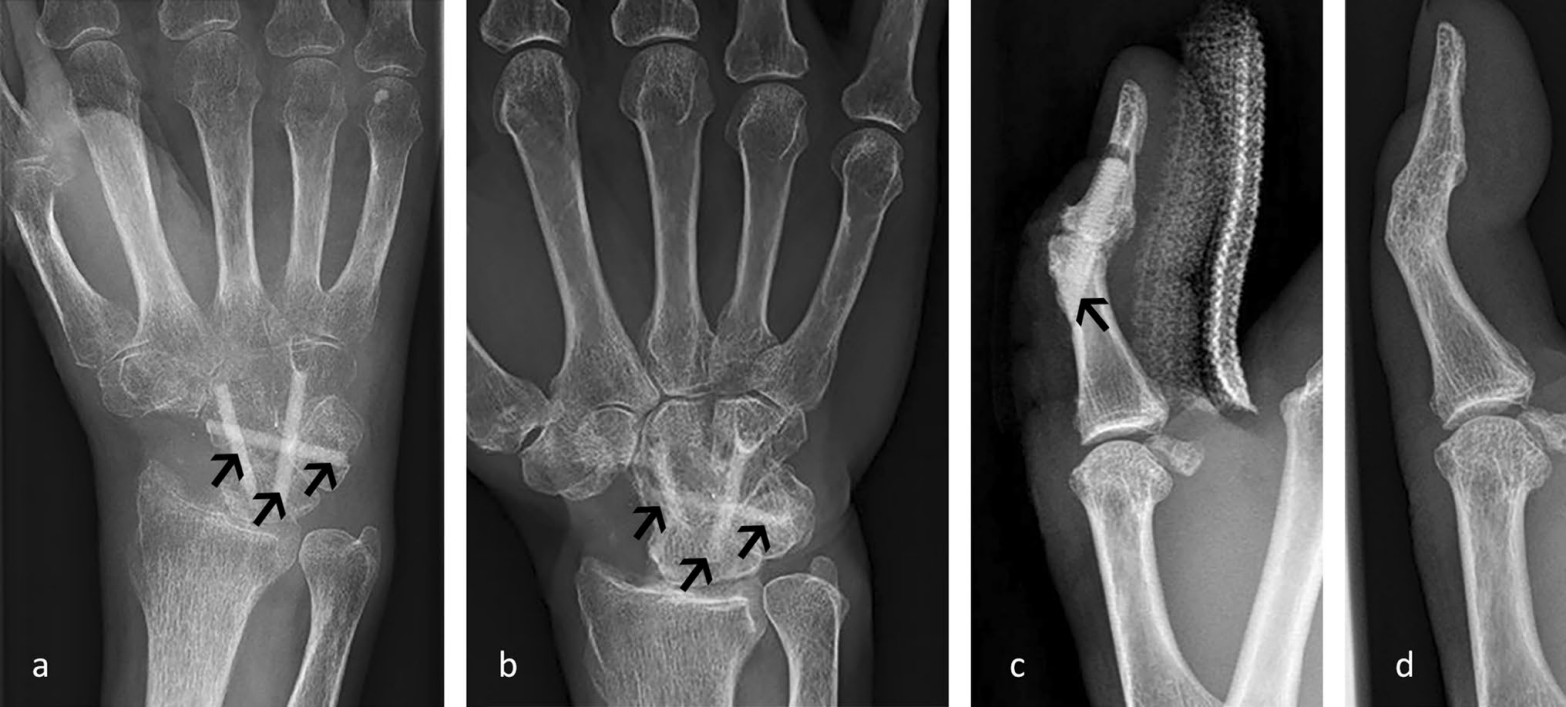
Examples of the application of the allogeneic screw in hand surgery. Postoperative radiographs showing the transplant used for a four-corner fusion **a** 10 weeks after surgery, **b** 20 months after surgery. Postoperative radiographs of an IP arthrodesis of the thumb **c** on the day of surgery, **d** 12 months after surgery. Transplants are marked by arrows

Figure 2 shows applications of allogeneic screws in hand surgery—a carpal bone fusion (four-corner fusion) (Fig. 2a, b) and a thumb interphalangeal (IP) arthrodesis (Fig. 2c, d). Bony consolidation of the arthrodesis and the incorporation and remodeling of the allogeneic screws is evident. The blurring shape of the transplants reveal an ongoing remodeling process (Fig. 2b) while in the case of the thumb IP arthrodesis the transplant is fully substituted by own bone material 12 months after surgery (Fig. 2d).

The majority of the patients did not show any complications in terms of wound healing, wound infection or soft tissue irritation. Complications occurred only in two cases: one patient developed a superficial wound healing disorder after a thumb interphalangeal (IP) arthrodesis, which healed without antibiotic therapy after two weeks. Another patient suffering from type 2 diabetes and peripheral arterial occlusive disease underwent surgery on the metatarsophalangeal (MTP) I joint to treat a painful arthrosis. Arthrodesis was achieved using two allogeneic screws (Fig. 1a). Postoperatively, a deep wound infection occurred including skin necrosis and a fistulous osteomyelitis. 8 weeks of antibiotic treatment and local wound surface treatment lead to complete healing of the osteomyelitis and joint fusion was achieved. In this case, the analgesic medication was longer (28 days) and pain sensation was higher compared to the values for analgesic medication and pain sensation collected for all other patients. Yet, the patient was free of pain 16 weeks after surgery. Radiology showed integration of the allogeneic screws and remodeling to endogenous bone 20 months after surgery (Fig. 1b).

**Fig. 3:**
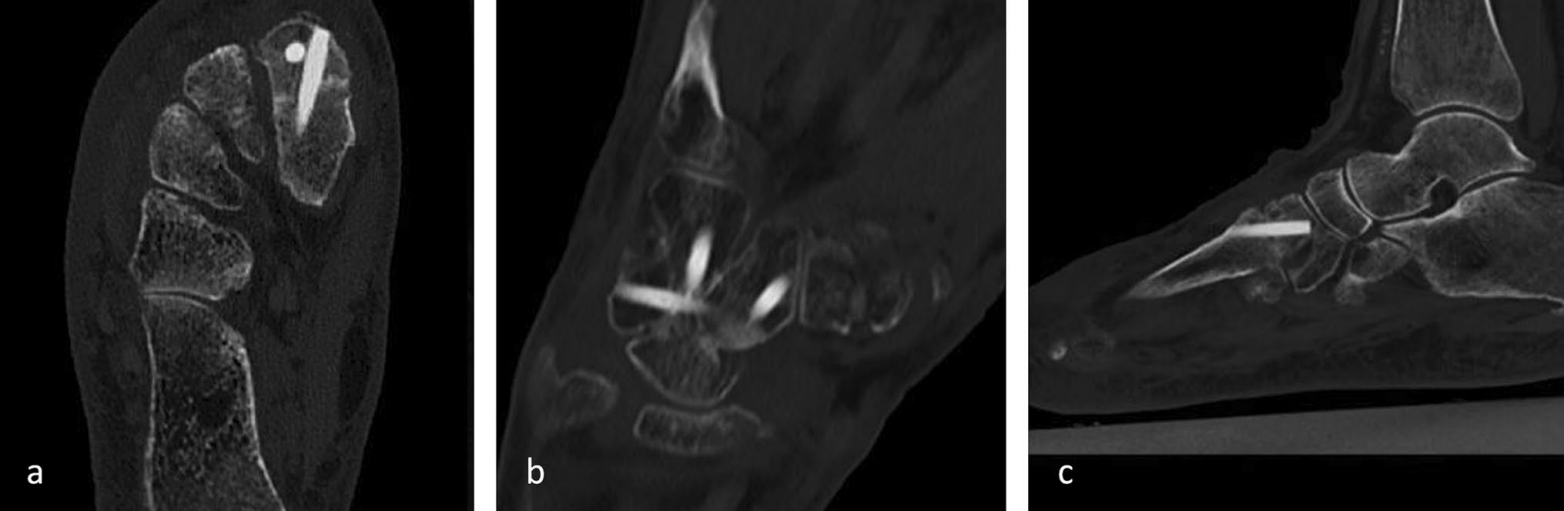
Computed tomography images of **a** a Lapidus arthrodesis 11 weeks after surgery, **b** a four-corner fusion 10 weeks after surgery and **c** a TMT II joint arthrodesis 6 weeks after surgery, all showing bony consolidation of the arthrodesis and the incorporation and partial remodeling of the allogeneic screw

According to radiology, full osseous fusion of the osteotomy or arthrodesis was seen for 31 patients (96.9%) at the last follow-up examination. Incipient osseous fusion was seen for one patient (3.1%). In six cases, osseous fusion was additionally determined by computed tomography (CT) 10–12 weeks after surgery. Figure 3 shows the CT images of a Lapidus arthrodesis 11 weeks postoperatively (Fig. 3a), a four-corner fusion 10 weeks postoperatively (Fig. 3b) and a tarsometatarsal (TMT) II joint arthrodesis 6 weeks postoperatively (Fig. 3c). In all three cases, the arthrodesis showed bony consolidation. Additionally, the CT confirms the osseous integration and partial remodeling of the transplants.

Neither nonunion nor failure of a allogeneic screw occurred in any of the reviewed cases. Hence, no revision surgery was performed.

Radiology revealed consolidation of the transplants in all cases. Radiolucent lines of more than 2 mm width paired with temporal dynamics were not detected in any case. In some cases, a radiolucent shade appeared between the transplant and the recipient bone six weeks after surgery. These lines result from an optical illusion known as Mach bands, where additional lines appear at the edges of different shades of grey [22]. At the second follow-up examination, the Mach bands have disappeared, as the density of the transplant has already been adapted to the density of the surrounding bone by physiological bone remodeling.

In two cases (6.3%), cystic lucency around the transplant was seen at the second follow-up examination. These signals were gone at the last examination. In no case sclerosis around the transplant pointing towards loosening of the screw or delayed incorporation was detected, thus ensuring constant rigidity of the osteosynthesis.

No signs of stress shielding were detected in any case.

In addition, the radiology review revealed that the period of remodeling of the allogeneic screw is not uniform. A fast remodeling process is seen for the Lapidus arthrodesis (Fig. 1c, d) and the thumb interphalangeal (IP) arthrodesis (Fig. 2c, d). In the case of the Lapidus arthrodesis, only a faint shade of one transplant was visible 12 months after surgery, the second transplant has been fully remodeled into own bone material, indicating an advanced remodeling process (Fig. 1d). Also for the thumb IP, the transplant had been fully substituted by own bone material after 12 months (Fig. 2d). In the case of the four-corner fusion (Fig. 2a, b), the transplants are still clearly visible 20 months after surgery, yet onset of remodeling can be seen (Fig. 2b).

## Discussion

The main aim of this study was to evaluate the efficacy of the transplant in stabilization of osteotomy and arthrodesis gaps to allow bone fusion and effective treatment.

Clinical and radiologic outcomes of the use of allogeneic screws in hand and foot surgical treatment are reported. Both, clinical (pain level, satisfaction, complications) and radiologic (consolidation, failure, osseous fusion) outcome was very good. In general, postoperative pain levels of the patients were low and the patient satisfaction was high. Wound healing disorder occurred in 2 out of 32 cases. In one case, a minor complication healed without intervention. In the other case, the patient’s medical history revealed an increased risk of healing disorders and postoperative complications.

In all cases, bony consolidation was achieved. Neither failure of an allogeneic screw nor nonunion occurred. X-ray and CT imaging confirmed incorporation of the transplant into the recipient bone without triggering bone tissue irritation. Potential graft rejection or allosensitization did not occur in any of the reviewed cases.

Similarly, reliable and effective defect filling capacity of allografts was recently reported for two-stage ACL revision surgery [23]. Yet, the grafts used in this study were cancellous grafts, whereas the bone screw derives from cortical bone.

This study analyzes the application of an allogeneic screw in a variety of indications in hand and foot surgery and thus allows a basic evaluation of the safety of the transplant and its ability to function as an osteosynthesis device leading to effective bone fusion and healing. Distal interphalangeal (DIP) joint arthrodesis is the most frequently performed procedure within this case series. Various treatment techniques including K-wire fixation, compression screw and plate fixation, bioabsorbable nail fixation or intramedullary elastic implant fixation are described [24–28]. Reported union rates vary in current literature and complication or revision rates are hard to compare [24, 26]. Union rates between 80 and 100% are reported for DIP arthrodesis using headless compression screws [25]. Treatment using the allogeneic bone screw seems to achieve results at similar levels. Yet, the case number is too low to draw reliable conclusions. Moreover, additional parameters such as functional scores are necessary to comprehensively compare various techniques. Analysis of the medical and economic impact of the allograft on particular indications compared to conventional treatment methods requires additional comparative studies.

Next to its reliable performance as an osteosynthesis device, various advantages of the allogeneic screw over conventionally used osteosynthesis systems are conceivable.

First, unlike metal devices, the allogeneic screw does not remain as a foreign matter within the bone and hardware removal is fully dispensable. Hardware removal belongs to the most frequent types of surgeries performed in orthopedics [16, 29] and represents a substantial economic burden for the health policy by causing direct costs for the health care system (e.g. costs of inpatient care, costs of surgery) and indirect costs due to postoperative nonproductive time of the patient. If hardware removal is necessary, the patient is again exposed to all surgical risks. Moreover, the risk of complications such as refracture, incomplete removal or wound infections persists [16, 18, 30].

Another advantage over metal devices is that the allogeneic screw is entirely placed within the bone without protruding elements. This explains the low level of postoperative pain and the smooth healing process with a low complication rate, as no irritation of the ambient soft tissue occurs.

Last but not least, metal implants are substantially stiffer than bone. This may lead to stress shielding and provoke disuse atrophy [31–34]. As the elastic moduli of the allogeneic screw and of the recipient bone are within the same range, no stress shielding is expected.

Compared to biodegradable osteosynthesis devices, the allogeneic screw is metabolized within the continuous bone remodeling process without the accumulation of degradation products. The degradation of bioabsorbable polymers in contrast triggers long lasting inflammatory processes. The former implant site may only be filled with bone once the implant material is fully degraded. This may lead to bone weakness during the degradation phase [35–37]. Complications arising from the use of bioabsorbable polymers include foreign matter reaction, osteolysis, synovitis, cartilage defects or local pain [38–43].

In the case of modern magnesium-based materials, the bone metabolism and the local immune reaction may also be favorably regulated by magnesium [44–46]. The drawback of these materials is a high corrosion rate which leads to extensive gas formation. Also, the alloying or coating components and the high concentration of local magnesium may have toxic effects [47, 48].

The problem of an unfavorable dissolution rate is automatically overcome in the case of an allogeneic screw as graft resorption and new bone formation are naturally balanced by the osteoclast/osteoblast activity.

After remodeling, the allogeneic screw is no longer detectable via X-ray. The duration of the remodeling process of the allogeneic screw depends on the specific perfusion and turnover rate of the recipient bone. A high turnover rate leads to faster transplant incorporation and remodeling. A contact area of maximum size between transplant and recipient bone ensures efficient incorporation of the transplant [49].

The available sizes of the allogeneic screw enable primarily the osteosynthesis of small bones. The treatment of fractures of large bones is not feasible due to the limiting length and diameter of the transplant which does not allow stable fixation of large bone fragments. Besides the application in arthrodesis and osteotomies in hand and foot surgery, use of the transplant in the treatment of small bone fractures, osteochondral defects and pseudarthrosis might be beneficial compared to current state-of-the-art treatment options. Moreover, the allogeneic bone screw might be beneficial for the use in pediatric orthopedics as metal removal is almost always necessary in the growing bone and the side effects of biodegradable materials are more critical in children. [45, 50] Additional studies are necessary to confirm this hypothesis.

This study has some limitations. The fact that surgeries, follow-up examinations, records review, and imaging evaluation have been performed by the same orthopedist carries the risk of introducing bias to the data, especially concerning the interpretation of radiology and CT images. Nevertheless, assessment of imaging immediately after surgery and at the follow-up examinations is usually done by the treating surgeon in collaboration with a radiologist, ensuring analysis of the images by at least two people. With a mean follow-up time of 1 year, the primary healing process and short-term outcome can be assessed. To evaluate additional parameters such as remodeling rates and possible long-term effects of the transplant, the observation period should span at least the turnover time of the transplant.

Detailed medical outcome and cost comparisons with conventional methods are not reasonable within the analysis of this case series due to the high variety of indications included and the even higher variety of alternative techniques and osteosynthesis systems available. Additionally, it may not be valid to simply compare the costs of the allogeneic bone screw to the costs of the available alternative devices. Also, the time of patient sick leave, time of hospital stay and needs of subsequent treatments must be taken into account to fully compare the economic impact.

In conclusion, the application of the allogeneic screw leads to low patient burden and fast recovery. The transplant is able to provide safe and mechanically stable bridging of bone defects. A high fusion rate combined with a low complication rate make the allogeneic screw a medically efficient osteosynthesis material.

Thus, the Shark Screw® transplant is a safe alternative to conventionally used fixation systems and suited for various kinds of osteosynthesis in hand and foot surgery.
